# Copper Zinc Sulfide (CuZnS) Quantum Dot-Decorated (NiCo)–S/Conductive Carbon Matrix as the Cathode for Li–S Batteries

**DOI:** 10.3390/nano12142403

**Published:** 2022-07-14

**Authors:** Thanphisit Artchuea, Assadawoot Srikhaow, Chakrit Sriprachuabwong, Adisorn Tuantranont, I-Ming Tang, Weeraphat Pon-On

**Affiliations:** 1 Department of Physics, Faculty of Science, Kasetsart University, Bangkok 10900, Thailand; thanidkai@hotmail.com; 2 Graphene and Printed Electronics for Dual-Use Applications Research Division (GPERD), National Security and Dual-Use Technology Center, National Science and Technology Development Agency (NSTDA), 112 Thailand Science Park, Phahon Yothin Road, Klong Nueng, Klong Luang, Phathum Thani 12120, Thailand; assadawoot.sri@gmail.com (A.S.); chakrit.sriprachuabwong@nectec.or.th (C.S.); 3 Department of Physics, Faculty of Science, Mahidol University, Bangkok 10400, Thailand; imingtang@yahoo.com

**Keywords:** lithium–sulfur batteries, quantum dots, conductive carbon materials, metal sulfides, sulfur host

## Abstract

Sulfur composites consisting of electrochemical reactive catalysts/conductive materials are investigated for use in lithium–sulfur (Li–S) batteries (LSBs). In this paper, we report the synthesis, physicochemical and electrochemical properties of CuZnS quantum dots (CZSQDs) decorated with nickel–cobalt–sulfide ((NiCo)–S)) mixed with reduced graphene oxide (rGO)/oxidized carbon nanotube (oxdCNT) (rGO/oxdCNT) ((NiCo)–S@rGO/oxdCNT) composites. These composites are for the purpose of being the sulfur host cathode in Li–S batteries. The as-prepared composites showed a porous structure with the CZSQDs being uniformly found on the surface of the rGO/oxdCNT, which had a specific surface area of 26.54 m^2^/g. Electrochemical studies indicated that the (NiCo)–S@rGO/oxdCNT cells forming the cathode exhibited a maximum capacity of 1154.96 mAhg^−1^ with the initial discharge at 0.1 C. The smaller size of the CZSQDs (~10 nm) had a positive effect on the CZSQDs@(NiCo)–S@rGO/oxdCNT composites in that they had a higher initial discharge capacity of 1344.18 mAhg^−1^ at 0.1 C with the Coulombic efficiency being maintained at almost 97.62% during cycling. This latter property is approximately 1.16 times more compared to the absence of the Cu–Zn–S QD loading. This study shows that the CuZnS quantum dots decorated with a (NiCo)–S@rGO/oxdCNT supporting matrix-based sulfur cathode have the potential to improve the performance of future lithium–sulfur batteries.

## 1. Introduction

The increasing demands for mobile energy storing devices require the development of new types of portable batteries [1,2,3,4,5]. Li-ion batteries (LIBs) have become one of the most generally used power storage devices. While LIBs were made for use in a variety of applications, batteries with higher energy densities are still needed. Thus, research has switched to finding new materials for use in lithium-ion batteries which could achieve this goal. Part of the focus of new research is on the development of lithium–sulfur batteries (LSBs). The reason for this is that the lithium (Li)–sulfur (S) batteries have a theoretical specific capacity of 1675 mAhg^−1^ and energy density of 2600 Whkg^−1^, which is more than 6–7 times higher than those of LIBs [4,5,6]. There are however several inherent properties of the materials in the Li–S batteries (LSBs) which have hindered the full commercialization of these types of batteries. First of all, both sulfur and lithium sulfides are highly reactive, leading to the formation of lithium sulfides Li_2_S_n_ (1 < *n* < 4), which is detrimental to the performance of the batteries. Several of these sulfides are insoluble in the solvent and they precipitate out and form as dendrite growth on the Li anode surface during cycling. This would result in declines in the performances of the batteries such as cycling stability, sulfur utilization and power storage capacity. To prevent the decline, many strategies were developed, such as new cathode designs [7,8,9,10], separator/functional interlayer fabrication [11,12,13] or organic liquid electrolyte [14,15]. For instance, one way to improve the cathode design would be to start with a matrix of a conductive carbon-based material (reduced graphene oxides (rGO), carbon nanotubes (CNT) or rGO/CNT onto which metal sulfides/oxides such as MnO_2_, CoS_2_, NiS_2_, MoS_2_ and FeS_2_ could be loaded onto [16,17,18,19,20,21,22,23,24,25,26,27,28,29]. In fact, the incorporation of these metals into reduced graphene oxide lithium sulfide batteries has become one of the more effective ways to enhance the energy and power densities and promote a strong reaction with lithium polysulfides. This would facilitate the redox kinetic of polysulfides and ensure a fast Li^+^ transport in the composite [26,27,28,29,30,31]. We focused on using the metal sulfides-based composites as the cathode for Li–S batteries. The binary metal sulfide ((NiCo)–S) in which NiCo_2_S_4_ was added has received widespread attention after it was seen to yield a richer redox reaction [32,33]. Earlier studies on the binary metal sulfide ((NiCo)–S) materials focused mainly on the synthesis steps needed to improve the inherently poor conductivity of NiCo_2_S_4_.

Future challenges to the development of the NiCo_2_S_4_-based composites for energy storage devices were reviewed by Ting-Feng Yi and coworkers [32]. Recently, Yanli Song et al. [33] showed that the cell with NiCo_2_S_4_ nanoparticles embedded in N-CNT as the sulfur host cathode material could deliver an average discharge capacity of 998 mAhg^−1^ at 0.1 C. They also studied how to preserve the excellent discharge capacity of 713 mAhg^−1^ at 1 C. All of this pointed to the superior reversible electrochemical property of NiCo_2_S_4_-based composite cathodes. However, the aggregation of the conductive carbon or metal sulfides/oxides which occurred in the composite had to be dealt with. The aggregation would reduce the number of active sites. This would restrict the utilization of active material in LSBs. To deal with the reduction of active sites, the design of hetero-structure catalysts with high catalytic performance had to be changed. The use of nanoscale-size materials (quantum dots (QDs)) was suggested and investigated [34,35,36,37]. QDs are very small particles (possessing unique quantum confinement properties) and have abundant active sites (huge surface-to-volume ratio) which lead to them being able to fill the open voids in the cathode materials. This allows them to efficiently tighten the adhesion of sulfur and provide the active sites with a means of anchoring the polysulfides.

Haochen Lu et al. [34], proposed that cobalt sulfide quantum dot embedded nitrogen/sulfur-doped carbon nanosheet composite materials (CoSx QD-NSC) be used as a polysulfide barrier in Li–S batteries. These cells would display significantly improved capacity, excellent rate capability and outstanding cycling stability. Xueliang Li et al. [35], studied the use of the CuS quantum dot modified carbon aerogel as a means to achieve high-performance lithium–sulfur batteries with a capacity of 1318 mAhg^−1^ at 0.2 C and long stability over 500 cyclings. Dong Cai et al. [36], demonstrated that the CdS quantum dot in a carbon nanotube induced the enhancement of the redox kinetics and suppressed the shuttle effect on the performance of Li–S batteries. Meanwhile, Daliang F et al. [37], designed a ZnS quantum dot multilayered N-doped carbon matrix (ZnS QDs@mNC) as a lithium battery. This matrix exhibited a high capacity of 1243 and 887 mAhg^−1^ at 0.1 mVs^−1^ for the discharge and charge capacities after the first cycle, respectively. Here forth, we used quantum dots of CuZnS nanocrystal-decorated nickel–cobalt–sulfide ((NiCo)–S)) mixed with a reduced graphene oxide (rGO)/oxidized carbon nanotube (oxdCNT) (rGO/oxdCNT) ((NiCo)–S@rGO/oxdCNT)-based composite material as the cathode in our LSBs. CuZnS is an alloy material composed of Cu_x_S and ZnS which are p-type inorganic materials with good electrical conductivity and a strong affinity for polysulfides [38]. A cell consisting of synthesized (NiCo)–S@rGO/oxdCNT combined with CuZnS QDs would be expected to be able to achieve greater utilization of the active material needed for converting the polysulfides and improving the electrochemical performance. As a result, using a (NiCo)–S@rGO/oxdCNT composite with sulfur loading as the cathode can create a Li–S battery with an initial discharge specific capacity (DSC) of 1154.96 mAhg^−1^ and enable a discharging capacity of 146.87 mAhg^−1^ after 100 cycles. The initial discharge capacity of 1344.18 mAhg^−1^ at 0.1 C achieved when CuZnS QDs are present is 1.16 times higher than when they are absent.

## 2. Materials and Methods

The preparation of the CuZnS quantum dot material (CZSQDs)-decorated nickel–cobalt–sulfide ((NiCo)–S)) loader reduced graphene oxide (rGO)/oxidized carbon nanotube (oxdCNT) (rGO/CNT) ((NiCo)–S@rGO/oxdCNT) composite-based sulfur cathode for performance lithium–sulfur batteries is divided into three parts. First, the oxidized CNTs are exposed to an acidic solution of sulfuric acid/nitric acid by sonication. This is followed by their assembly on rGO (which acts as the conductive carbon matrix). Next, the hydrothermal method is used to synthesize the (NiCo)–S@rGO/oxdCNT matrix. Finally, preparation of CuZnS QDs-decorated (NiCo)–S@rGO/oxdCNT matrix is accomplished by self-assembly method.

### 2.1. Synthesis of Oxidized CNT (oxdCNT)

Oxidized CNTs were obtained by a mild acid oxidation treatment. Briefly, 0.3 g of commercial CNT were suspended in a mixed aqueous solution of H_2_SO_4_ (3M) (QRëC 98%, Auckland, New Zealand) and HNO_3_ (3M) (Lobe Chemie, Mumbai, India) in a 3:1 volume ratio. After stirring at 60 °C for 15 min, the suspension was sonicated for two hours in a conational ultrasonic bath (100 W, 40 kHz, Manufacture expert). Then, the oxidized CNT was diluted with 250 mL deionized water and vacuum filtered and rinsed with DI water until the pH of the filtrate became neutral. Then they were freeze-dried to obtain oxdCNT.

### 2.2. Synthesis of CuZnS Quantum Dots (CZSQDs)

The synthesis of CuZnS QDs is carried out according to the literature [39]. Briefly, a mixture of CuCl_2_·2H_2_O (1 mmol) and ZnCl_2_ (1 mmol) powder was dissolved in a mixed solution of oleylamine (4.5 mL), 1-octadecans (4.5 mL) and oleic acid (1 mL) in three-necked flask at a temperature of 260 °C under N_2_ atmosphere for 10 min. Another solution of thioacetamide (sulfur source) (3 mmol) dissolved in 3 mL of oleylamine was injected into the Cu and Zn solution. The reacted solution remained there in the flask for another 5 min before being removed and heated to room temperature. Then, the obtained colloidal CuZnS QDs were separated by mixing with methanol at the ratio of 1:20 *v/v* and centrifuged. The resultant CuZnS QDs were dried under vacuum for 3 h, enclosed by aluminum foil and kept refrigerated for further use.

### 2.3. Synthesis of (NiCo)–S@rGO/oxdCNT Matrix

The (NiCo)–S@rGO/oxdCNT matrix was prepared using a modified hydrothermal method. Typically, a suspension containing 90 mg of oxdCNT and 270 mg of rGO (oxdCNT:rGO = 1:3 *wt*/*wt*) in a mixture of 3 mL NH_4_OH, 90 mL DI water and 90 mL ethanol was stirred at 60 °C for one hour. Then, 2 mL mixture solution consisting of Ni(NO_3_)_2_·6H_2_O (0.2 M), Co(NO_3_)_2_·6H_2_O (0.4 M) and Na_2_S·9H_2_O (0.8 M) was added to the above solution and stirred for an hour. After that, the solution was transferred to a Teflon-lined stainless-steel autoclave and hydrothermally treated at 160 °C for 12 h. After cooling down to room temperature and washing with DI water and ethanol, the solid products of (NiCo)–S@rGO/oxdCNT were freeze-dried at −40 °C for 8 h. Meanwhile, the (NiCo)–S@rGO/CNT (non-acid treated CNT) composite was synthesized in similar procedure but with untreated CNT as a starting material.

### 2.4. Preparation of CuZnS QDs-Decorated (NiCo)–S@rGO/CNT Composite and Sulfur Composite

The synthesized QDs-decorated (NiCo)–S@rGO/oxdCNT matrix was prepared by self-assembly method in which the CuZnS QDs powder was dissolved in 50 mL of hexane in a necked flask and mixed for 10 min. This was followed by the addition of the as-prepared (NiCo)–S@rGO/oxdCNT where the weight ratio between QDs and matrix was kept at 3:7. The solution was stirred at 80 °C for 5 h and then vacuum freeze-dried to obtain the CuZnS QDs-decorated (NiCo)–S@rGO/oxdCNT powder CZSQD@(NiCo)–S@rGO/oxdCNT). For sulfur composite, the CuZnS QDs-decorated (NiCo)–S@rGO/oxdCNT/S powder and sulfur powder in a 1:3 mass ratio were ground in agate mortar for 30 min followed by the melt diffusion method at 155 °C for 12 h and cooled to room temperature. The absence of the CuZnS QDs loading was prepared by the same method. For the purpose of the capability of the synthesized samples, the synthesis route of composites was synthesized using the same procedure.

### 2.5. Characterization of the Materials

The morphologies of the as-prepared samples were imaged using scanning electron microscopy (SEM) and transmission electron microscopy (TEM) (JEM-3010, JEOL, Tokyo, Japan). The crystal structures of the synthesized powders were determined by powder X-ray diffraction (XRD) (Bruker diffract meter, Model D8 Advance) using the Cu Ka radiation and operating at 40 kV with 40 mA current. The XRD patterns were scanned from 2θ = 10°–70° at a scanning speed of 1 s per step with an increment of 0.037° per step. For the FT–IR absorption measurements of the rGO/oxdCNT and the (NiCo)–S@rGO/oxdCNT powders were used. The powders were mixed with KBr and pressed into pellets under a pressure of 10 tons for 1 min. The pellets were analyzed using an FT–IR spectrophotometer (Spectrum GX, Perkin Elmer, Waltham, MA, USA) which performed 16 scans over the range 370–4000 cm^−1^. The specific surface areas were determined using the nitrogen adsorption/desorption tests (JW-BK11) after the samples were degassed at 100 °C overnight and were calculated using the conventional Brunauer–Emmett–Teller (BET) method. To determine the surface chemical composition of the as-prepared samples, X-ray photoelectron spectroscopy (XPS) (K-Alpha 1063, Thermo Fisher Scientific was used. The content of sulfur in the CZSQDs@(NiCo)–S@rGO/oxdCNT/S composite was confirmed using thermo gravimetric analysis (TGA, Perkin-Elmer) in a nitrogen atmosphere. The working electrode was prepared by mixing active materials, acetylene black, and polyvinylidene fluoride (PVF) in a weight ratio of 80:10:10 with N-methylpyrrolidone (NMP) used as the solvent. The slurry was coated onto aluminum foil and dried in vacuum at 60 °C for 12 h. The obtained film was cut into disks with diameters of 14 mm (electrode thickness was about 20 µm) with the mass of the active material being 5 mg. The electrolyte used was 1 molL^−1^ lithium bis (trifluoromethane sulphonamide (LiTFSI) in 1, 3 dioxolane/1, 2-dimethoxyethane (DOL/DME) (1:1 *v/v* with 2 wt% LiNO_3_ used as an additive). This additive plays the role of suppressing the redox shuttle of the dissolved LiPSs on the Li anode. The amount of electrolyte was 80 µL in each coin cell. CR2025 coin-type cells were assembled in an Ar-filled glove box with the metal lithium being used as the anode. Polypropylene microporous films (Celgard2400) act as separators. The galvanostatic charge and discharge tests were determined with a NEWARE testing instrument in a voltage range between 1.7–2.8 V versus Li+/Li ratios at the various current density. For the electrochemical measurements, an electrochemical workstation (CHI660E, Shanghai Chenhua Corp., Shanghai, China) was used for cyclic voltammetry (CV) testing, the voltage range was set to 1.8 and 2.8 V (vs. Li^+^/Li) and the scanning rate was 0.1 mV/s. Electrochemical impedance spectra (EIS) tests were carried out with the AC amplitude being 5 mV and the frequency range being 100 kHz to 0.01 Hz.

## 3. Results and Discussion

### 3.1. Physico-Chemical Properties of the As-Prepared rGO/oxdCNT, the (NiCo)–S@rGO/oxdCNT and the QDs Loading Materials

**Figure 1:**
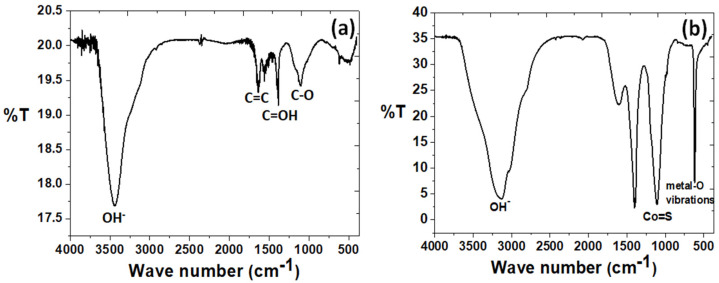
FT–IR spectra of rGO/oxdCNT (**a**) and (NiCo)–S@rGO/oxdCNT (**b**) powders.

We observe a reduced agglomeration of the reactive products because of the presence of the functional groups of −COOH or −OH on them since they have become negatively charged due to the effects of the ionization process. These negatively functional groups act as the nucleation sites for the metal cations (Ni^2+^ and Co^2+^). This is caused by the electrostatic interactions between them and the carbon matrix. During the hydrothermal treatment, the (NiCo)–S particles will grow on the surfaces of the rGO/oxdCNT matrix. The presence of the functional groups on the as-prepared samples can be seen in the FT–IR results (See Figure 1). The FT–IR spectra of rGO/oxdCNT are shown in Figure 1a. The broad absorption band at 3400 cm^−1^ is attributed to the O–H stretching vibration of adsorbed water molecules [24,25]. The peaks at 1213 cm^−1^, 1384 cm^−1^ and in the range of 1500–1600 cm^−1^ are due to the C–O stretching, the C=OH stretching and the sp^2^ hybridized C=C in-plane vibrations, respectively [22,23]. The strong peak at 1082 cm^−1^ in the (NiCo)–S@rGO/oxdCNT composite shown in Figure 1b corresponds to the blending vibration of sulfurated groups and could also be due to the Co–S stretching indicating the presence of Ni–Co–S in the synthesized composite [25].

**Figure 2:**
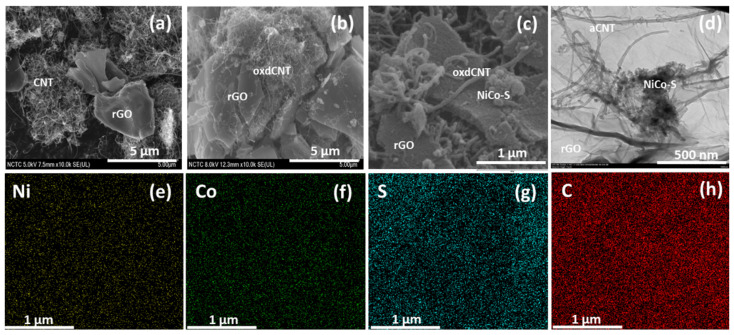
SEM images of rGO/CNT (**a**), rGO/oxdCNT (**b**) and (NiCo)–S@rGO/oxdCNT composite (**c**). (**d**) TEM image of (NiCo)–S@rGO/oxdCNT composite and corresponding EDS elemental mappings (**e**–**h**).

To investigate the morphologies of the as-prepared substrates, SEM images were taken. Figure 2a–c show the SEM images of the rGO/CNT (untreated CNT) substrate, the rGO/oxdCNT (acid-treated CNT) substrate and the substrate after (NiCo)–S metal oxide loading, respectively. From the SEM image, the low interconnection between rGO and CNT can be seen in the rGO/CNT composite (Figure 2a). In Figure 2b (for rGO/oxdCNT), we see a homogenous composite matrix with a well-interconnected network of the rGO and the oxdCNT. In Figure 2c, which is of the (NiCo)–S@rGO/oxdCNT substrate, the formation of smaller metal sulfide particles in the rGO/oxdCNT matrix can be seen. The elemental mappings of the (NiCo)–S@rGO/oxdCNT composites are shown in Figure 2e–h. They exhibit the characteristic peaks of the elements Ni, Co, S, and C, indicating that these elements are in the sample. The TEM images of the substrates clearly show the deposition of the (NiCo)–S particles which are well attached to the rGO/oxdCNT matrix (Figure 2d). This is beneficial for promoting the redox reaction in Li–S batteries.

**Figure 3:**
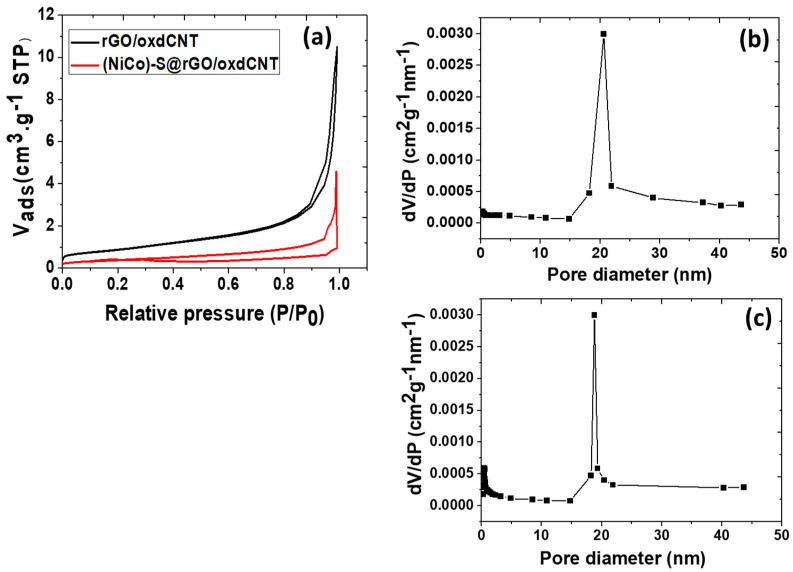
Nitrogen adsorption–desorption isotherms of rGO/oxdCNT and (NiCo)–S@rGO/oxdCNT (**a**). Pore-size distributions of rGO/oxdCNT (**b**) and (NiCo)–S@rGO/oxdCNT (**c**) powders.

To study the porosity of the as-prepared samples, the nitrogen adsorption/desorption isotherm measurement was performed (See Figure 3a). The BET surface areas of the rGO/oxdCNT and (NiCo)–S@rGO/oxdCNT powders were 32.56 and 29.96 m^2^/g with an average pore diameter of 20.45 and 16.53 nm (corresponding to mesopores) (Figure 3b,c), respectively. The reduction of surface area can be ascribed to the appearance of metal sulfides in the rGO/oxdCNT matrix.

**Figure 4:**
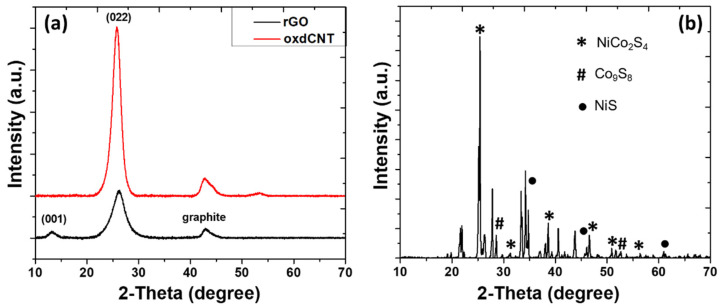
XRD pattern of the rGO and oxdCNT (**a**) and (NiCo)–S@rGO/oxdCNT powders (**b**).

**Figure 5:**
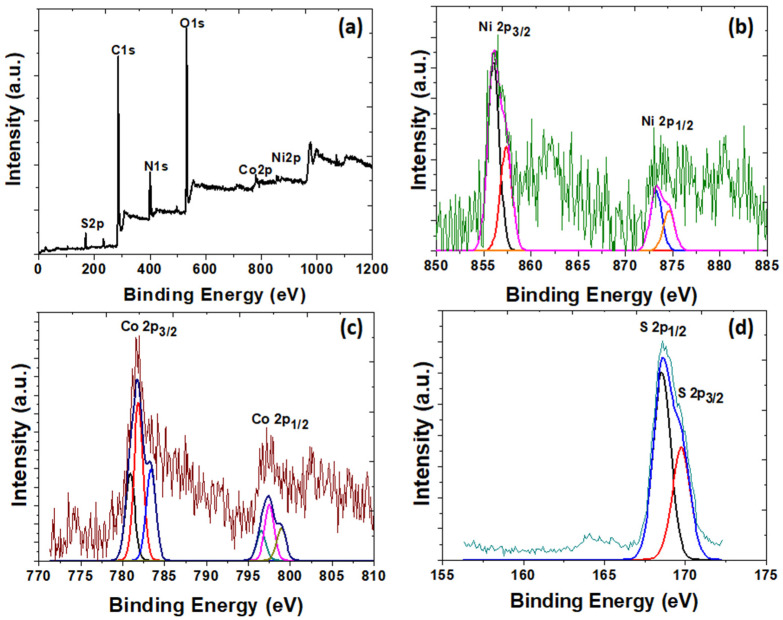
XPS spectra of the as-prepared (NiCo)–S@rGO/oxdCNT composite: (**a**) survey spectra, (**b**) Ni 2p, (**c**) Co 2p and (**d**) S 2p.

The crystalline nature of oxdCNT, rGO and (NiCo)–S@rGO/oxdCNT were investigated by XRD. As seen in Figure 4, the diffraction peaks at 2θ ≈ 10.27 and 26.1° originate from the rGO [20]. A strong diffraction peak of functional CNT was formed at 2θ ≈ 25°. The low-intensity peaks were formed at 2θ ≈ 32.5° and 43° can be attributed to the graphite (Figure 4a). It was also observed in the XRD patterns (Figure 4b) that the dominant phase formed was NiCo_2_S_4_ (PDF 020-0782) as evident by the appearance of the characteristic peaks at 2θ = 26.7°, 31.6°, 38.3°, 47.5°, 50.7° and 55.3°, which correspond to (220), (311), (400), (511), (440), and (731) planes, respectively [24,29]. The three minor peaks at 2θ = 29.8°, 47.5° and 52° can be assigned to be the (311), (511) and (411) planes of Co_9_S_8_ (PDF 073-1442). In addition, the characteristic diffraction peaks of the NiS phase (PDF 020-1280) well defined at 2θ = 34.7°, 46°, 53.5°, 62.7° and 65.4° correspond to the (101), (102), (110), (200) and (201), respectively [40]. To determine the chemical composition of the as-prepared, XPS measurements were carried out (Figure 5a–d). The XPS survey spectra (Figure 5a) show the presence of the elements C, O, Ni, Co, S and N. The spectra of Ni2p (Figure 5b) can also be fitted with two spin doubles at 856.02 eV and 873.21 eV due to 2p_3/2_ and 2p_1/2_ electrons, respectively, corresponding to the characteristics of Ni^3+^. These are typical of surface oxidation [41]. In the Co2p spectrum (Figure 5c), the peak located at 781.89 eV is due to 2p_3/2_ and that at 796.55 eV is due to 2p_1/2_ electrons and is characteristic of ion Co^2+^ [30,31]. The peaks in the S2p spectrum (Figure 5d) can be fitted with the binding energy at 168.58 eV and 169.74 eV, which are due to the S 2p_1/2_ and S 2p_3/2_, respectively [30,31]. According to the XPS analysis, the metal elements in the (NiCo)–S particles are mainly divalent electrons.

**Figure 6:**
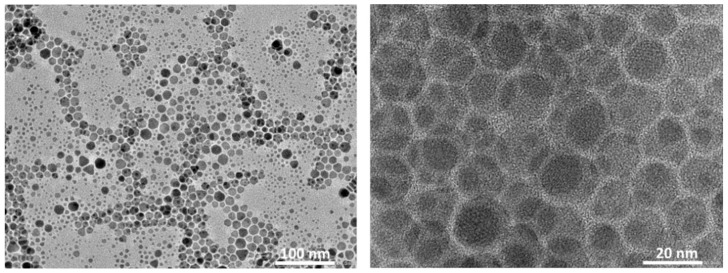
TEM images of CuZnS QDs at low (**left**) and high (**right**) magnification.

The morphology of CuZnS QDs was also examined by TEM (Figure 6). The synthesized particles are seen to be evenly dispersed without any aggregations. The as-prepared particle sizes of CZS QDs are between 10 and 20 nm. The morphologies and microstructures of the sample after the CuZnS QDs were decorated on their supporting matrix are investigated by the SEM, TEM, EDS and BET techniques.

**Figure 7:**
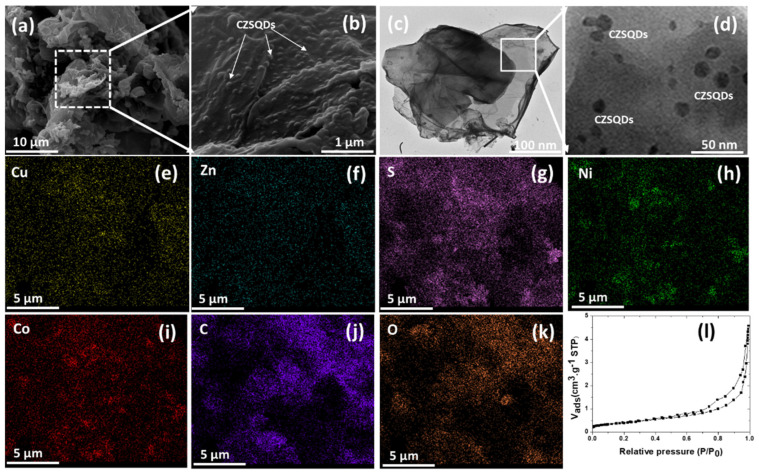
SEM images (**a**,**b**) and (**c**,**d**) TEM image of CuZnS QDs-decorated (NiCo)–S@rGOoxdCNT supporting matrix. EDS elemental mappings (**e**–**k**). (**l**) Nitrogen adsorption–desorption isotherms.

**Figure 8:**
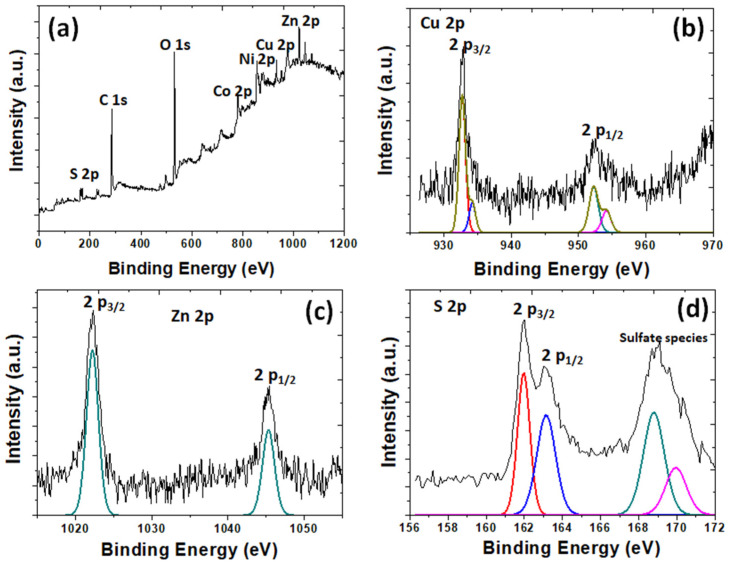
XPS spectra of the as-prepared CuZnS QDs decorated (NiCo)–S@rGO/oxdCNT composite: (**a**) survey spectra, (**b**) Cu 2p, (**c**) Ni 2p and (**d**) S 2p.

Figure 7a shows the SEM image of CuZnS QD-decorated (NiCo)–S@rGOoxdCNT supporting matrix. Metal sulfide particles were to be deposited on the porous network of the carbon matrix. To monitor this, images of the composites were taken and are shown in Figure 7b), where uniform smaller sizes of CZSQD are seen to be highly dispersed on the carbon matrix. This suggests that the decorated CZSQDs are the favored formation on the supporting surface. The micro/nanostructure of the CZSQDs@(NiCo)–S@rGO/oxdCNT composite powder was investigated using TEM imaging. Figure 7c shows the assembled sheet-like graphene and the carbon nanotube with QDs. The high-resolution TEM (Figure 7d) clearly shows the CuZnS QDs decorated on the rGO sheet with the particle size being about 10–20 nm. From the elemental mapping (Figure 7e–k), it was found that elements Cu, Zn, S, Ni, Co, C and O are mainly distributed over the whole Cu–Zn–S QDs and (NiCo)–S particles. To confirm the porous structure of the CuZnS QD-decorated (NiCo)–S@rGOoxdCNT supporting matrix, the specific surface area was determined by BET. Figure 7l shows the N_2_ adsorption–desorption isotherms. The BET analysis gives the surface area of 26.54 m^2^g^−1^. This specific surface area is slightly lower than that in the absence of QDs, i.e., in the (NiCo)–S@rGOoxdCNT supporting matrix. The lower value may be due to a better distribution of the CuZnS particles and to the lack of accumulation of these particles on the matrix. The structure of the QD-containing composites would allow accelerated ion transport. The better distribution of active materials would enhance the rapid redox on the surface of the active materials. The surface chemical composition of CZSQDs@(NiCo)–S@rGO/oxdCNT was determined by XPS (Figure 8). A typical spectrum of the as-prepared sample shows the presence of Cu, Zn, S 2p, Ni 2p, Co, C 1s and O species (Figure 8a). The presence of the CuZnS QDs was calibrated by referencing the Cu (Figure 8b) at 944.7 and the signal at 962.6 eV can be assigned to be Cu(II) in the structure of CuZnS [38]. The spectrums of Zn 2p (Figure 8c) at 1045.3 eV and 1022.2 eV are due to Zn 2p_1/2_ and to Zn 2p_3/2_ of Zn^2+^ [38]. The two peaks at 161.2 and 162.3 eV (Figure 8d) are due to S 2p_3/2_ and S 2p_1/2_ of sulfur, respectively [42]. In addition, the binding energy at about 169 eV is due to the oxidized sulfur caused by the exposure of the sample to the air atmosphere [43].

**Figure 9:**
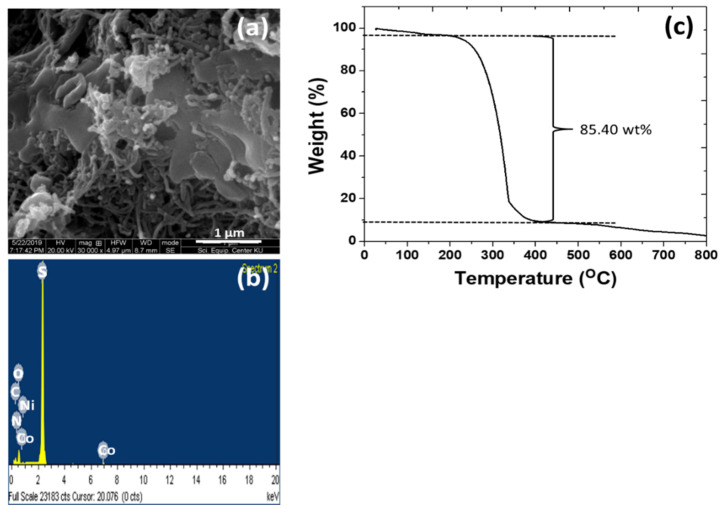
SEM image/EDS analysis (**a**,**b**) and Thermogravimetric curves of the S-loading CuZnS QD-decorated (NiCo)–S@rGO/oxdCNT composite (**c**).

After adding the S, the lamellae become thicker and the particles become larger (Figure 9a). The element mappings (Figure 9b) of the composite contain the main characteristic peak of the S element. This indicates a successful loading of sulfur into the CuZnS QD-decorated (NiCo)–S@rGO/oxdCNT composite. Based on the TGA curve (Figure 9c), the thermal decomposition of the substrate occurs when the sulfur in the substrate begins to evaporate between 200–400 °C. The evaporation is seen in the weight loss of approximately 85.40 wt% above these temperatures.

### 3.2. Electrochemical Performance

**Figure 10:**
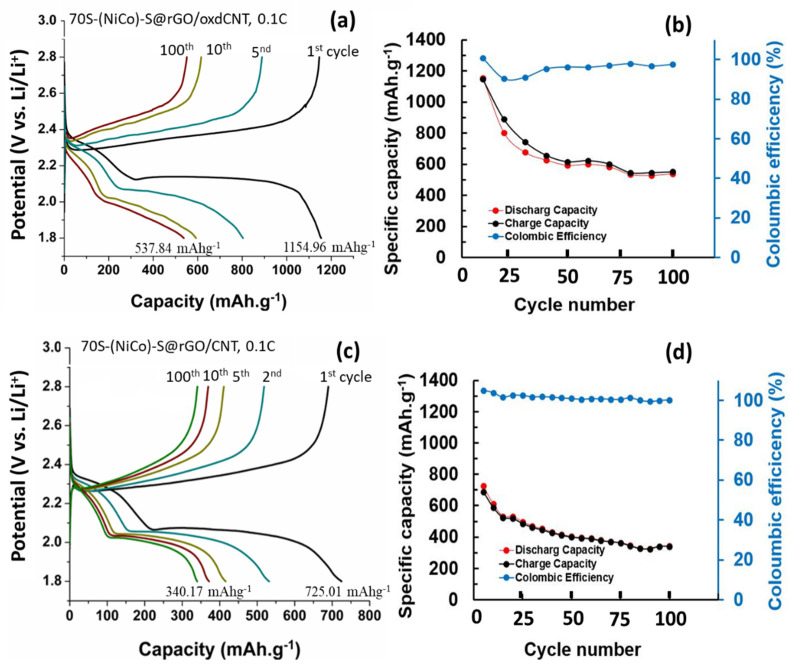
(**a**,**b**) charge/discharge curves, cycle performance and Coulombic efficiency of (NiCo)–S@rGO/oxdCNT/S. (**c**,**d**) Charge/discharge curves and Coulombic of nonacid treated CNT ((NiCo)–S@rGO/oxdCNT/S) at the current density of 0.1 C at 1st, 2nd, 5th, 10th and 100th cycles.

**Figure 11:**
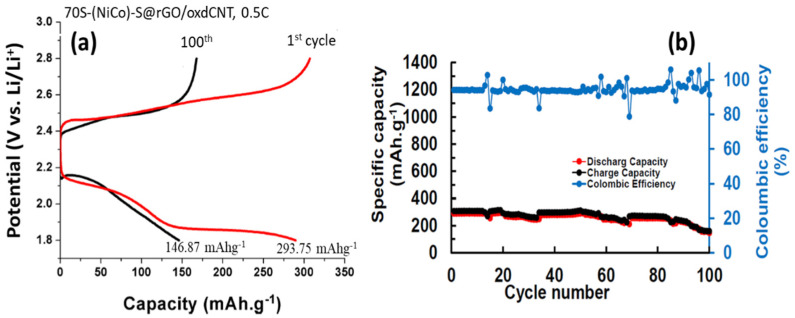
(**a**,**b**) charge/discharge curves, cycle performance and Coulombic efficiency of (NiCo)–S@rGO/oxdCNT/S at the current density of 0.5 C at 1st and 100th cycles.

Figure 10 and Figure 11 show the electrochemical performance of the lithium–sulfur cells made with (NiCo)–S@rGO/oxdCNT. The galvanostatic charge–discharge curves cycling at a current density of 0.1 C are shown in Figure 10a. The CV plots show plateaus at approximately 2.3 V and 2.1 V during discharge. The plateau at 2.3 V is due to the opening of the S_8_ ring while that at 2.1 is due to the reduction of the long-chain polysulfide ions Li_2_S_n_ (4 ≤ *n* ≤ 8) [29,30,31]. The chain Li_2_S_n_ is formed during the reaction [S_8_ + Li^+^ + e^−^ → Li_2_S_n_ (4 ≤ *n* ≤ 8)]. The plateau arises during the reducing Li_2_S_n_ to Li_2_S_2_/Li_2_S [Li_2_S_n_ + Li^+^ + e → Li_2_S + Li_2_S_2_]. Figure 10b compares the cycling performances of the S-loaded (NiCo)–S@rGO/oxdCNT at 0.1 C for 100 cycles. The capacity of 1154.96 mAhg^−1^ is obtained after the 1st initial discharge cycle (higher compared with that non-acid treated CNT (725.01 mAhg^−1^) (Figure 10c,d). The capacity gradually decreased to 537.84 mAhg^−1^ after 100 cycles. The (NiCo)–S@rGO/oxdCNT (with acid-treated CNT as an electrode) exhibits a higher capacity (~1.67 times) compared with that of (NiCo)–S@rGO/CNT (without acid-treated CNT as the electrode). This behavior is connected to the homogenous composite matrix which exhibits a well-interconnected network of the rGO and the oxdCNT as seen in the morphology analysis (Figure 2a–c). In addition, the incorporation of oxdCNT can effectively prevent restacking within the graphene sheets. This leads to the porous conductive network needed for fast charge transfer and increases the porosity needed for rapid diffusion of active ions which would enhance the electrochemical performance. Noticeably, when the cycle is increased, the Coulombic efficiency is maintained at almost 97.62% during the cycling. This is attributed to the stability of the (NiCo)–S@rGO/oxdCNT composite-based S cathode. To observe the rate capability, the effect of increasing the current rate from 0.1 to 0.5 C was investigated. As can be seen, the (NiCo)–S@rGO/oxdCNT composite shows decreased capacities in which the initial discharge capacity decreased from 1154.96 mAhg^−1^ (for the current density of 0.1 C) (Figure 10a,b) to 293.75 mAhg^−1^ (Figure 11a,b). The capacity after 100 cycles is significantly lowered with this change, i.e., the capacity is reduced to 146.87 mAhg^−1^).

**Figure 12:**
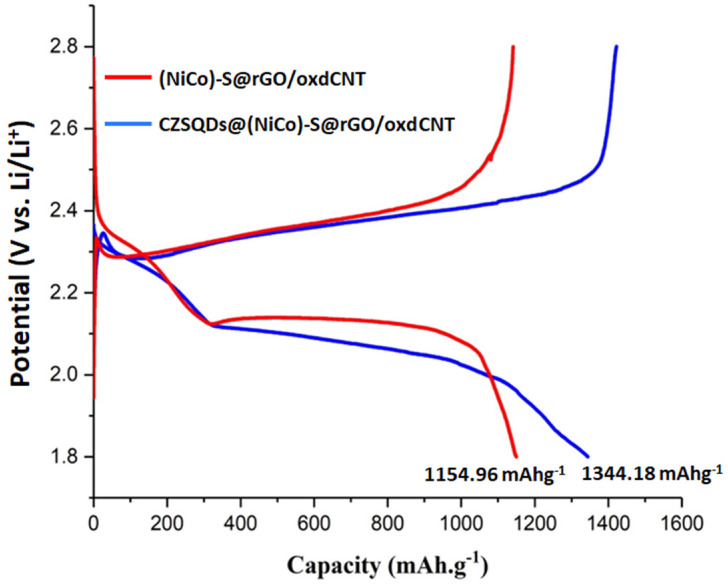
First initial charge/discharge curves with and without CuZnS QDs decorated (NiCo)–S@rGO/oxdCNT/S at the current density of 0.1 C.

The electrochemical performance of the CuZnS QD-decorated (NiCo)–S@rGO/oxdCNT matrix is shown in Figure 12. As can be seen, both with and without the CuZnS QDs exhibit significantly better performance in the initial charge/discharge capacities. The initial discharge of CZSQDs@(NiCo)–S@rGO/oxdCNT is 1344.18 mAhg^−1^ at 0.1 C, which is approximately 1.16 times more compared to that of an absent CuZnS QD-loaded supporting matrix of nickel–cobalt–sulfide with rGO/oxdCNT. As seen in Figure 12, the initial discharge exhibited two plateaus which are observed at around 2.39 V and 2.03 V corresponding to the reduction of the cyclo-octasulfur (S_8_) to a long chain soluble polysulfide and to the decomposition of the long-chain polysulfide to insoluble Li_2_S_2_/Li_2_S [29,30,31].

**Figure 13:**
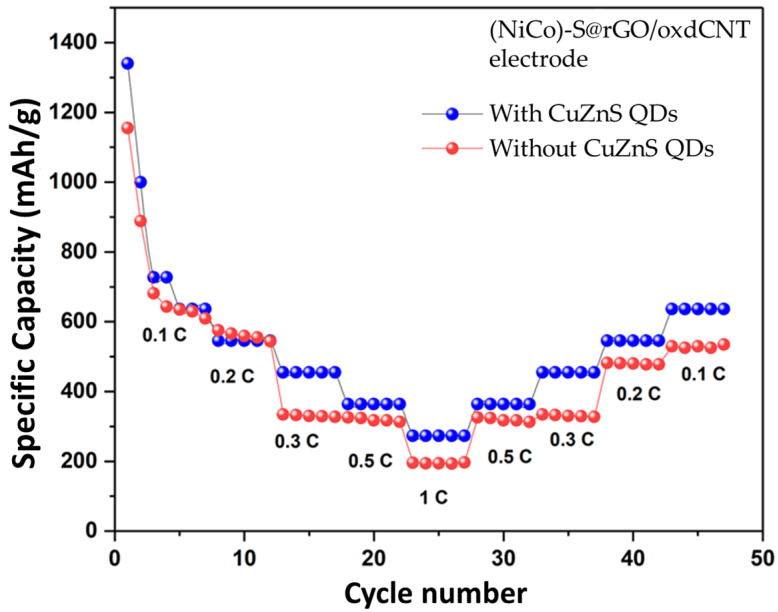
The rated capacity of Li–S batteries with and without CuZnS QDs decorated (NiCo)–S@rGO/oxdCNT electrode.

**Figure 14:**
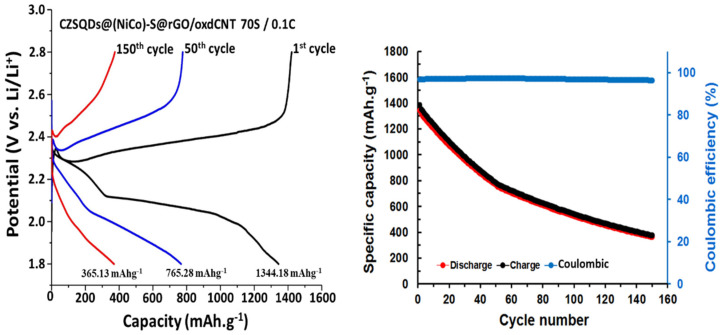
Charge/discharge curves, cycle performance and Coulombic efficiency of CZSQDs@(NiCo)–S@rGO/oxdCNT/S at the current density of 0.1 C at 1st, 50th and 150th cycles.

The lower anodic peak and the higher catholic peak of CuZnS QD-decorated (NiCo)–S@rGO/oxdCNT compared with those without the CZSQDs demonstrated the enhancement of electrochemical activities and the lowering of the energy barriers for the onset of the redox reactions [36]. These phenomena can be associated with the intrinsic positive effects of the CuZnS QDs which advance the development of the electrochemical conversion in the sulfur-based active species. The rate capabilities of the battery with the CuZnS QD-decorated (NiCo)–S@rGO/oxdCNT electrode was further tested (Figure 13). The reversible discharge capacities of this electrode at 0.1, 0.2, 0.3, 0.5 and 1.0 C rates are 636, 545, 454, 363 and 272 mAhg^−1^, respectively. When the current rate reversed back to 0.1 C, the reversible capacity of 636 mAhg^−1^ was achieved which is higher than that without the CuZnS QD-decorated (NiCo)–S@rGO/oxdCNT electrode (534 mAhg^−1^). The result indicated that the incorporated CuZnS QDs improved rates and pronounced catalytic site capability in the stage charge–discharge process. To further investigate, we looked at the charge/discharge capacity along with the cycle performance of the CZSQDs@(NiCo)–S@rGO/oxdCNT as the Li–S cathode. As seen in Figure 14 for the cyclic performance recorded at 0.1 C, the 1st initial discharge had a capacity of 1344.18 mAhg^−1^, which after 150 cycles, dropped to 365.13 mAhg^−1^ with a Coulombic efficiency of 99.08%. The lower discharge capacity of the cell after the first few cycles is proposed to come from the low stability of the loading of active materials in the heterostructure during the discharge process. This is attributed to the irreversible deposition of sulfur species outside of the CZSQDs@(NiCo)–S@rGO/oxdCNT.

Based on the fact the Coulombic efficiency is maintained at almost 100%, which is higher compared that with without the CuZnS QDs loading (97.62%), indicates that the presence of the CuZnS QD-decorated (NiCo)–S@rGO/oxdCNT supporting matrix can effectively enhance the redox reactions and reduce the polysulfide shuttle (enhance sulfur utilization).

**Figure 15:**
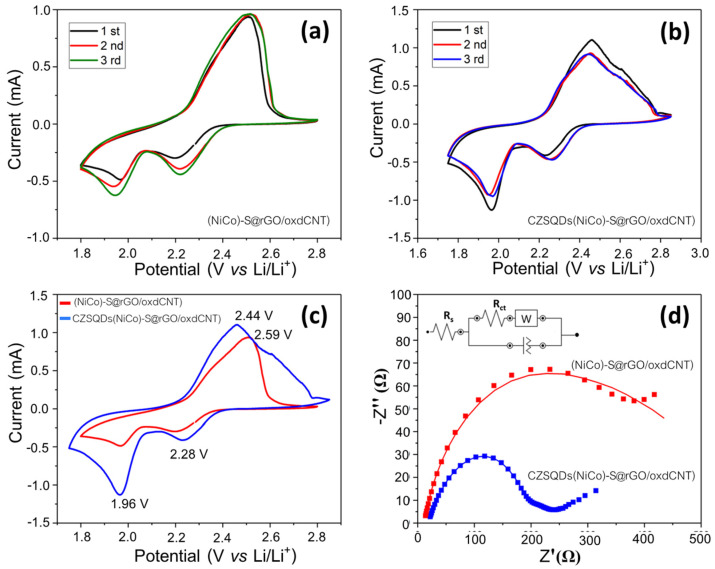
CV curves for the cells with (**a**) and without (**b**) CuZnS quantum dot (CZSQDs) decorated-(NiCo)–S@rGO/oxdCNT for the first three cycles at 0.1 mVs^−1^. (**c**) comparison of CV curves and electrochemical impedance spectra (EIS) (**d**).

To demonstrate the critical role of CuZnS quantum dot (CZSQDs)-decorated (NiCo)–S@rGO/oxdCNT as the sulfur host cathode in the performance improvement, the CV curves were measured. These are displayed in Figure 15a,b. For the batteries with and without the CuZnS quantum dots loading, the CV curves for the two types of Li–S batteries exhibit slight changes in the current and in the potential shifts after the first to third cycles suggesting quiet catalytic stability of the cells. The CV curves for the two types of batteries almost overlap each other, indicative of high electrochemical reversibility. As seen in the CV curves of both types of cells in the potential range of 1.8–2.8 V, the cell with CuZnS quantum dots exhibits two reduction peaks at around 2.28 and 1.96 V in the cathodic scan, with the peak currents of 0.5 and 1.10 mA, respectively. Meanwhile, the cathode without the CuZnS quantum dots shows slightly lower reduction peak potentials at around 2.22 and 1.94 V with lower peak currents of 0.34 and 0.56 mA, respectively. These can be attributed to the formation of long-chain lithium polysulfides (Li_2_S_4–8_) and short-chain Li_2_S_2_/Li_2_S, respectively [44]. The oxidation peak centered at 2.4 V with the peak current of 1.0 mA could also be attributed to the reversible processes of transformation from Li_2_S_2_/Li_2_S to polysulfides and finally to S_8_ [26,44,45].

Compared to the sulfur host with CuZnS quantum dots and without the quantum dots (Figure 15c), CV curves for both types of cells show a slightly (but obvious) shift in the reduction peak. The cell with quantum dots (CZSQDs(NiCo)–S@rGO/oxdCNT) is positively shifted, indicating that the CZSQD loading could improve the polarization of the cell. This positive shift can be attributed to the fact that the CZSQD loading causes effective adsorption which in turn leads to a catalytic effect on the conversion of polysulfides [44]. Additionally, the intensity of the reduction peaks of the cells with CZSQD loading is higher than those of the cells without the quantum dots suggesting the stronger polysulfides adsorption ability, which results in a higher degree of the reducing reactions. Considering the oxidation peak potentials, the cells with CZSQDs@(NiCo)–S@rGO/oxdCNT as the cathode have a distinguishable negative shift which could be attributed to the fact that they facilitate the oxidation of short-chain to long-chain LiPS. This improves the S utilization and increases the capacity of the charging process [44].

Figure 15d shows the EIS spectra of the cells with and without CZSQD loading. The EIS profiles display a semicircle in the high- and middle-frequency ranges and an inclined line in the low-frequency range, which demonstrated similar reaction processes for the batteries with and without CZSQD loading. The batteries based on CZSQDs@(NiCo)–S@rGO/oxdCNT showed nearly interfacial resistance (*R_s_*) (19.4 Ω) compared with those of the (NiCo)–S@rGO/oxdCNT (11.3 Ω). Meanwhile, the charge transfer resistance (*R_ct_*) of the cells with CZSQD loading (187 Ω) was lower than that of the cathode without the CuZnS quantum dots (364 Ω). The smaller *R_ct_* demonstrating the CZSQDs@(NiCo)–S@rGO/oxdCNT possesses a kinetically favored conversion reaction with rapid lithium ions and electrons transport.

## 4. Conclusions

We presented an efficient synthesis of a (NiCo)–S@rGO/oxdCNT composite and CuZnS QDs which can be used to decorate the composite to obtain a sulfur-based cathode material that could improve the performance of lithium–sulfur batteries (LSBs). The CZSQDs@(NiCo)–S@rGO/oxdCNT composite has a porous structure in which the quantum dots and metal sulfides are evenly dispersed and confined uniformly within the rGO/oxdCNT matrix. The porosity of the surface of the CZSQDs@(NiCo)–S@rGO/oxdCNT substrate allows for 85.40 wt% sulfur loading. This results in the creation of pathways for rapid charge transfer and ion diffusion and enhances the performance of the discharge capacity up to as high as 1344.18 mAhg^−1^ after the initial first discharge cycle. This is a significant improvement in the initial capacity when compared to that of discharge capacity with a cathode made from (NiCo)–S@rGO/oxdCNT (1154.96 mAhg^−1^). The positive electrochemical performance when using the CuZnS QD-decorated supporting matrix confirms that the QDs allow for excellent interaction between the cathode material and the soluble polysulfide species created after the Li_2_S_n_ phase conversion. Their inclusion will also boost the redox kinetics of chemicals in the cathode system. The positioning of the sulfur, the long-term stability, the type of quantum dots, and the other materials in the cathode supporting matrix should be further studied in order to achieve a better discharge-specific capacity.

## Data Availability

The datasets generated during the current work are available from the corresponding author on reasonable request.
